# Political determinants of digital health: beyond the rainbow

**DOI:** 10.1093/heapro/daaf014

**Published:** 2025-04-03

**Authors:** Sara L M Davis

**Affiliations:** Centre for Interdisciplinary Methodologies, University of Warwick, Coventry, CV4 7AL, United Kingdom

**Keywords:** digital health, political determinants, global health governance

## Abstract

In 2021, the World Health Organization issued its first global strategy on digital technologies and health, calling on member states to develop their own national digital health strategies. However, the normative tools that guide the development of these strategies do not prompt member states to consider how broader political factors, such as law and policy, governance, and civic engagement, could shape health outcomes in the digital age. The digital gender divide, algorithmic biases linked to race, transnational private sector actors, and more must be thoroughly addressed in future digital health strategies. Experiences from the global HIV response have demonstrated that it is possible to do this, by mobilizing diverse government and non-governmental actors to systematically assess and act to strengthen the legal and political environment for health. This essay draws on the HIV response to critically engage with recent literature on the digital determinants of health, proposing an approach to analyzing broader political determinants of health, including commercial determinants of health, and other laws, policies, governance, and civic engagement relevant to digital health strategies. By rendering visible the role of politics, governance, and civic engagement in digital health, strategies can be tools to mobilize broad collaborations and advocacy that creates an enabling environment.

**Contribution to Health Promotion**

The legal and political determinants shaping the digital transformation of health include not only direct regulation of health and information and communication technology but also broader political factors that are both local and global.
The political determinants of digital health include national and international law and policy; local and global governance; and civic engagement; as well as the influence of transnational private sector actors.
The experience of the HIV sector offers useful lessons and tools that can contribute to a practical approach.

## INTRODUCTION

In 2021, the World Health Organization (WHO) launched the first *Global Strategy for Digital Health 2020-2025.* (WHO defines ‘digital health’ as including ‘digital applications and services used to aid individuals, the health workforce, and health system users, to address challenges and improve access to quality information and health interventions’ (WHO 2023, v).) Coming one year into the COVID-19 pandemic, the strategy’s approval by the World Health Assembly marked a growing awareness among health officials that the digital transformation was radically, and likely permanently, reshaping how people accessed health information and services.

This transformation would require a new approach to counter the growing fragmentation among diverse public and private actors. By developing national digital health strategies, states were encouraged to ‘integrate leadership, financial, organizational, human and technological resources’ and establish ‘the basis for a costed action plan which enables coordination among multiple stakeholders’ to meet shared objectives (WHO 2021, 15, 21).

WHO’s *National eHealth Strategy Toolkit* (WHO and ITU 2012, aka ‘the Toolkit’) is one of the normative tools that has since been used to guide the development of national digital health strategies, and it is emblematic of both the strengths and limitations of the current approach. At its time of publication, in 2012, the toolkit was an important step forward, in that it brought together health and information and communication technology (ICT) governance for joint analysis and planning.

The Toolkit does call for strategies to consider ‘aspects such as governance, policy, legislation, standards and human resources’ (2012, 5). However, these aspects are defined narrowly, with reference mostly to regulation of the formal health and ICT sectors. The guiding questions for use in strategy development consider such policy areas as data protection, e-commerce, and access to information (2012, 61–65). They do not include, to choose a few examples, questions about the digital gender divide, human rights, or the digital inclusion of diverse groups, such as persons with disabilities, older persons, young people, or ethnic and linguistic minorities.

It is perhaps unsurprising, then, that many digital health strategies do not consider these questions either. A recent review of 20 national digital health strategies found that all 20 failed to mention or address gender inequalities, and that most also did not consider human rights or ‘access issues, biases, discrimination, and other needs of diverse populations’ (Serra et al. 2024, 4). The broader academic literature has been slow to come to grips with human rights in the digital transformation of health, too: a systematic review of digital health studies in Southeast Asia finds human rights underexplored in the literature (Poulsen et al. 2024).

In this article, I propose that the development of national digital health strategies should begin to stretch beyond the regulation of the formal health and ICT sectors, to consider other political determinants of digital health. These include intersecting areas of law and policy, governance, civic engagement, and the potential impact of transnational commercial interests that shape access to, uptake of, and design of digital health.

This proposed approach is inspired by approaches and tools used with some success in the global HIV response. The global HIV response has included, arguably, the best-resourced global effort to address the political factors shaping health access and outcomes. As discussed below, diverse actors (UN agencies, government agencies, donors, academics, private sector, and civil society) have come together to coordinate analysis of political determinants of HIV, and have successfully coordinated collaborative and pragmatic action to change the political and structural environment in order to benefit health.

The political challenges in the digital transformation of health are certainly different from the challenges raised by the HIV response, but both have been global in scope and thus demand creative thinking, new analytical tools, and new collaborations. Health and ICT officials who lead the development of future national digital health strategies cannot be expected to directly solve all the political and structural factors that shape the design of, access to, and usage of digital technologies and platforms for health. However, the national digital health strategies they develop should, at a minimum, not ignore those broader structural factors, and ideally should consider them with targeted objectives and plans. The processes for development of national digital health strategies offer opportunities for creative thought, learning, convening, and consultation with potential new governmental and non-governmental partners who can bring their expertise to the table, and help to create more comprehensive digital health strategies that guide implementation into the next stages of the digital transformation.

This essay begins by considering the current literature on digital determinants of health with a critical lens. It then considers related work on political determinants of health, and suggests some ways in which to apply this thinking to inform digital health strategies.

## THE DIGITAL DETERMINANTS OF HEALTH

Discussions about the need for more robust digital health governance have gained urgency and importance in the past 5 years, as the digital transformation has taken off and created an increasingly complex and dynamic online ‘health information ecosystem’ (Purnat 2024). In particular, scholarly attention has increasingly focused on how digital technologies, artificial intelligence (AI), and digital spaces have become drivers or ‘digital determinants’ of health. The phrase ‘digital determinants’ was first proposed in a major review of digital health governance by the *Lancet/Financial Times Commission on Governing Health Futures 2030: Growing Up in a Digital World*, which argued, based on this review, that digital technologies had become not just tools, but actually ‘new determinants’ of health outcomes (Kickbusch et al. 2021).

This conceptualization of ‘digital determinants’ has since enjoyed widespread acceptance in academic global health literature. Scholars have used digital determinants to, for example, discuss the influence of racialized and other biases in shaping the design and application of digital technologies for health (Charpignon et al. 2023, Fraser et al. 2023), or to analyze inequalities in access to and effective use of digital health technologies, and more (Kaihlanen et al. 2022; van Kessel et al. 2022). In 2022, Jahnel and colleagues integrated digital determinants into the widely used socioecological rainbow model of determinants of health inequity.

While the rainbow helpfully illuminates the proximity of related determinants; as a metaphor, it does not leave much visual space for politics. In the past several years, evidence only continues to accumulate that the inequalities raised in studies of the digital determinants of health noted above (e.g. unequal access, algorithmic biases) manifest differently across populations based on age, gender, education, race and ethnicity, language, geographic location, and socio-economic status (Ferraris et al. 2023; Szinay et al. 2023). While a rainbow shows colors that are non-intersecting, in reality, intersecting factors such as race, class, gender, and other structural inequalities can and do shape access and uptake to digital health. The rainbow thus falls short as a representation of the complex ways in which humans increasingly interact with engineered systems (Özdemir 2019). Existing forms of systemic discrimination against groups based on gender, age, race, disability, or other status can produce biased data that reaffirms multiple intersecting human biases when fed into algorithms, and could, for example, result in misdiagnosis or poorer quality of medical care (Wójcik 2022). For those who are transgender or gender-nonconforming, digital health can offer new access to information and services, but binary systems for gender identification can also create problems when health information management system design fails to include diverse options (Costanza-Chock 2020; Radix et al. 2022).

Thus, broader inequalities can interact with digital tools and platforms directly to hamper access to digital determinants of health. These broader inequalities also amplify multiple intersecting forms of digital exclusion, when lack of digital inclusion in turn impacts ‘access to the wider determinants of health, such as housing or employment opportunities, [which may] become dependent on digital access routes’ (Honeyman et al. 2020, 1).

Such inequalities are certainly social. They can also be political: produced by policy, law, political rhetoric, elections, and other political tools and actions.

Any analytical optic and its graphic representation will have strengths and limitations, but the moment may have come to step back and consider whether the conceptual clarity offered by ‘digital determinants of health’ might also be limiting in some ways. If we focus too much on the gatekeeping role of the digital, is there a risk that it may flatten out and obscure other dimensions of the commercial and political forces and structures that promote and shape the digital determinants of health? Looking beyond the rainbow, who or what determines the digital determinants? Or put more simply, if digital technologies are the drivers of health inequity, then who is behind the wheel?

In order to explore political determinants of health in the digital age further, and consider how national digital health strategies might disarticulate and address them systematically, we turn to the literature on the political determinants of health, and to research and tools from the HIV sector that may be applicable to digital health strategy development.

## POLITICAL DETERMINANTS OF (DIGITAL) HEALTH

Determinants have tended to multiply in academic global health literature, and there has been a certain amount of conceptual fluidity in the way different determinants of health are discussed and applied. In particular, political and commercial determinants have sometimes been used in overlapping ways. In this section, we briefly touch on two overlapping and related literatures on political and commercial determinants, and then propose an approach to thinking more systematically about political determinants of digital health.

In her initial essay, calling for attention to the political determinants of health, Kickbusch (2005) argued that global health needed to begin to consider political factors, such as the international system of finance and trade, and their impact on health, particularly for persons living in poverty. Scholars have since built on Kickbusch’s groundbreaking essay to discuss political determinants in diverse ways: for instance, to describe the impact of populist politicization on health systems during the COVID-19 pandemic (Dickman and Chicas 2021) ; or the state’s use of health policy as a political tool in China, where officials used COVID-19 tracking apps to isolate and detain critics of the national pandemic response (Ip 2021). Erlangga and colleagues (2023) have called policy a political determinant of health financing, which in turn shapes health outcomes. Some consider the direct, malicious targeting of health systems by armed actors in conflict as a political determinant of health (Kennedy et al. 2017, Macak et al. 2020).

About 10 years later, in their call to unite diverse critical analyses of the power of the private sector under a shared banner, Kickbusch et al. (2016) made the case for a new type of determinant, the ‘commercial determinants of health’, using this to describe the powerful influence of commercial actors on the regulatory environment, and the harmful effect of this on health (for instance, on the spread of non-communicable diseases). In this framing of commercial determinants as ‘strategies and approaches used by the private sector to promote products and choices that are detrimental to health’, politics appears to be somewhat subsumed into the category of the commercial.

However, the political determinants of health have maintained their appeal as an analytical lens, and in a 2022 special issue of *Global Policy*, Storeng and colleagues (2021) in turn called for greater attention to the political determinants of digital health, arguing that the concentration of power in private actors had accelerated ‘the private capture of global public health’. They proposed that consideration of political determinants of inequality in the digital transformation of health in low- and middle-income countries (LMIC) center the influence of commercial actors on the policy environment, in order to understand how their influence may result in a poorly regulated market-driven digital health sector (Storeng et al. 2021). The COVID-19 pandemic, they noted, sparked billions of dollars of new investment in mobile health and COVID-19 management apps, as well as health information management systems. The pandemic also marked the first widespread use of social media to seek and share health information, producing newly viral ‘infodemics’ of misleading information (WHO 2024).

The unchecked power of these transnational private actors, Storeng and colleagues (2021) warned, posed significant challenges for national health officials. For example, the rise of web platforms and social media has positioned these platforms as knowledge brokers for health information. Google now commands over 81% of global searches (Bianchi 2024), so answers to search questions by anyone seeking health information online—as millions of people now do daily––are shaped by ‘surveillance capitalism’, in which advertising-driven priorities and the logics of search engine optimization monetize intimate personal information and shape the prioritization of search results (Eysenbach and Kohler 2003, Zuboff 2019).

Thus, a simple answer to the question, ‘who is behind the wheel’ of digital determinants of health could therefore be ‘the private sector’; again, political and commercial determinants seem to be subsumed into the same analysis.

However, another answer to the ‘who is behind the wheel’ question could consider the role of evolving and fluid systems of national and global governance in either constraining or failing to constrain, these private sector actors. In addition to the commercial forces shaping digital health governance, I argue, there are other areas of politics at play as well, that should be considered alongside the commercial influences.

In his study of the passage of President Obama’s health policy in the United States, Dawes (2020) helpfully proposes to group political determinants of health into three categories: **law and policy, governance**, and **civic engagement**. In considering how each of these categories might apply to digital health, I suggest we consider the commercial determinants as a cross-cutting aspect of political determinants which should be considered by those developing national digital health strategies as one influential political factor—but not the only such aspect.

***1. Law and policy***—In her 2023 report to the UN Human Rights Council on *Digital Innovation, Technology and the Right to Health*, UN Special Rapporteur on the Right to Health Tlaleng Mofokeng observes that ‘the speed of the digital transformation has outpaced the ability of States to effectively safeguard human rights’ (UN Office of the HIgh Commissioner for Human Rights (OHCHR) 2023). In particular, building on the human right to health, her report drew attention to the accessibility, availability, acceptability, and quality of health information and services to propose a normative framework for analysis of the right to health in relation to digital innovation and technology. Mofokeng called for states to establish robust laws and policies to ensure that digital technologies uphold the right to health, and noted initiatives to develop new norms, guidelines, and policies at the regional and UN levels; she also rightly noted that this work is currently at an early stage of development.

Thus, analysis of the impact of laws and policies on health to inform national digital health strategies should consider the following:

- *Systemic legal inequalities*—When considering the impact of the digital transformation, digital health strategies should include consideration of the broader legal and political environment relevant to gender equality, racial/ethnic/linguistic inequality; rights of sexual minorities, persons with disabilities, migrants, persons living with HIV, indigenous persons, and any other protected statuses. For example, criminalization of HIV transmission and discrimination against persons living with HIV might make persons living with HIV reluctant to disclose their status or seek information about treatment online, for fear of being exposed to negative judgment. Likewise, in Kenya, key populations (specifically, men who have sex with men, sex workers, and people who use drugs) mobilized to block national health agency efforts to gather their biometric data (such as fingerprints) as part of an HIV study, out of concerns that this data could be used to target them for arrest (Davis and Maleche 2018). In these examples, a policy environment that disfavors one or another population could undermine digital trust and willingness to participate in digital health.
- *Regulation of digital access to the social determinants of health (aka the ‘digital welfare state’)*—Developers of digital health strategies and interventions should consider how digital access, inclusion, and literacy may become facilitators or barriers to other services, including education, housing, social protection, and welfare; or what former UN Special Rapporteur on Extreme Poverty and Human Rights Philip Alston (UN Human Rights Office 2019) called the ‘Digital Welfare State’. As Alston notes, the growth of the digital transformation means that citizens increasingly require technology and Internet access in order to access the social determinants of health, such as education, housing, employment, and social protection. These in turn are also determinants of health; so regulation of the digital welfare state indirectly shapes health outcomes.
- *Regulation of transnational tools, apps, and platforms—*Given that the most widely-used health apps, wearables, or other technologies used for health in a given country may be operating well outside the formal health system, and may also be produced and profiting companies overseas, national digital health strategy development should also identify which of those tools, apps, or technologies are widely used in the country, and consider whether the existing regulatory framework effectively addresses both the quality of these tools, and effective governance of the data they produce.

In order to identify clear associations between specific laws and policies and related health outcomes, Mackenbach argued for the use of political epidemiology: ‘creating opportunities, either by design or in the analysis, for identifying causal effects of political variables (structures, processes, outputs) on population health’ (2014, 2). In particular, Mackenbach calls for consideration of ‘laws, taxes, social security benefit, public services, etc. that will ultimately produce the health and other societal outcomes of interest’ (2014, 2).

A related field of legal epidemiology has since advanced the scientific study of health impacts of law (Poirier et al. 2022). Methods have further been proposed for comparative country policy surveillance, arguing for the need, for example, to assess the impact of pandemic control measures in order to inform future policy (Burris et al. 2021).

In the future, research could study the relationship between a variety of specific laws and policies, such as those listed above, and related health outcomes.

***2. Governance***: Political determinants of health also go beyond what Burris called ‘laws on the books’ to encompass what governments, courts, police, and others do in practice. Important streams of work on political determinants of health have considered how good (or poor) governance can shape health outcomes. Examples of this include analyses of structural racism, such as the review currently led by the *O’Neill-Lancet Commission on Racism, Structural Discrimination and Global Health* (Erondu et al. 2023).

In the context of digital health, even where the legal and policy environment is favorable, it is important to consider how well existing governance procedures work to implement them and ensure accountability. For example, are national offices adequately resourced to implement laws and policies that protect health in the digital age?

If access to smartphones, airtime and wifi are required to access health information, appointments, or telemedicine, then existing gaps in digital infrastructure (for instance, lack of Internet or mobile phone coverage for those living in rural areas) may have a significant impact on health outcomes in particular groups that have less socio-economic resources to pay their way online, including women and marginalized groups. Digital health strategies should consider who might be impacted by a lack of wifi or airtime, and how to mitigate the risk of their digital exclusion.

Are inequalities not formally codified in law shaping access, inclusion, and experience of end users? For instance, are gender inequalities shaping the ability of women and girls to access health information and services, if they are required to share phones with male family members? Are linguistic diversity in the population being effectively addressed with existing digital health information systems and tools?

Likewise, are there government strategies in place led by other government agencies beyond ICT and health, or initiatives led by municipal or local government, or initiatives led by professional associations, that might complement the national digital health strategy and provide good practices for digital inclusion? These might include national strategies or plans to address the diverse needs of women, youth, persons with disabilities, or ethnic or national minorities that have experienced historical discrimination.

In the process of developing national digital health strategies, digital health strategy developers could consider mapping out good practices that exist on a small scale in the country to address digital divides, such as innovative approaches to data governance led by indigenous groups or national minorities; community-led platforms and groups on social media; or partnerships between local government with civil society or community-led networks that foster digital inclusion and digital trust with marginalized groups.

***3. Civic engagement***: In considering digital determinants of health, we also need to consider the role of human agency. Visually, in the social determinants rainbow, humans appear somewhat overwhelmed by the powerful forces shaping health beyond their control. However, the digital determinants of health are not a naturally occurring phenomenon: they have been manufactured by individuals and groups, for profit, quite recently. Unlike rainbows, the digital turn is something that humans have produced and can potentially shape, through voting or collective action.

Some scholars have previously considered civic engagement as a political determinant of health: Atti and Gullis propose voting for specific candidates as a political determinant of health, given decisions those officials could make about health policy if elected (Atti and Gulis 2017). Syed and colleagues (2022) argue that gerrymandering of voters could affect health policy decisions. In the case of the U.S. movement to pass President Obama’s health policy, Dawes notes, grassroots activism was a cross-cutting political determinant that influenced voting, governance, and policy (2020).

Likewise, three decades of the global HIV movement have demonstrated the power of civic engagement to influence the political determinants of health, as activists and institutions have drawn on human rights norms, scientific evidence, and community mobilization to make their case, countering government denialism and successfully arguing for interventions and resources for the HIV response (Chan 2015).

In digital health governance, the role of civic engagement has yet to emerge as a significant political determinant; but efforts are underway to mobilize greater collaboration in this area by civil society groups, including through such convenings as RightsCon, an annual hybrid meeting of government officials, rights advocates, business leaders, and others on the intersection of human rights and technology (RightsCon 2024).

To facilitate civic engagement at local and transnational scales, advocates will need new information and skills. Digital health literacy has to date been understood as knowledge used to access reliable health information, but van Kessel and colleagues (2022) suggest it is now becoming a ‘super determinant of health’ that impacts other determinants. In the future, national digital health strategies should consider more the need to define and promote digital health literacy, including digital human rights literacy and health data governance literacy, in order to equip individuals and groups with the knowledge and skills to demand the kind of digital health governance they need in order to fulfill their human right to health (Davis et al. 2023; Digital Health and Rights Project Consortium 2024). This could include scientific knowledge (about the technology, data, and AI); legal and rights knowledge (about laws and policies on data, digital governance, and AI governance); and advocacy knowledge (how to analyze power, mobilize community power to advocate for effective change, and how to use evidence and media to make the case).

Expanding these forms of digital health literacy would enable civic engagement and links among movements, including transnationally, that could create the momentum for greater participation in multi-stakeholder governance for digital health.

At the same time, critics have noted that multi-stakeholder governance platforms have often been coopted by private sector interests; they have underscored the need to attend to inequalities in governance platforms that may marginalize critical voices from decision-making, with attendant risks of ‘ethics-washing’ and tokenism (Manahan and Kumar 2021). Similarly, Wong and colleagues (2021) warn of the risk of ‘museumization’ of youth inclusion in digital health governance. In the Digital Health and Rights Project, an international academic and civil society consortium in which I participate, civil society and youth leaders are holding periodic reflection discussions on their experiences of participation in global and national consultations, with the aim of identifying structures, principles, and practical approaches that facilitate meaningful participation (as opposed to tokenism).

## AN ENABLING ENVIRONMENT FOR DIGITAL HEALTH: TOOLS AND STRATEGIES FROM THE HIV SECTOR

All three domains discussed above––law and policy, governance, and civic engagement––are overlapping, interrelated, and mutually reinforcing. Dawes’ analysis re-situates human agency at the center of political determinants. He argues that voting and civic engagement put in place decision-makers who create and execute policy; government provides a mechanism for decision-makers to keep, enforce, or change the status quo; while policy ‘essentially concretizes or codifies the final decision or action’ (Dawes 2020, 47).

Currently, these three categories of political determinants are not routinely integrated or considered in digital health strategy development. However, there are approaches and tools that have been developed to support such systematic consideration in the HIV response. Significant work has been done by lawyers, policymakers, UN officials, health officials, civil society, and community leaders to develop systematic analytical tools to assess what constitutes an ‘enabling legal and policy environment’ for the HIV response, considering such political determinants as criminalization of HIV transmission, criminalization of key populations affected by HIV, access to medicines, gender inequalities, and levels of funding for community-led responses to the HIV epidemic.

The Global Commission on HIV and the Law, hosted by the UN Development Programme, was one such example. The Global Commission convened legal and policy reviews on related themes and also held open hearings in different geographic regions that brought together government officials, experts, and community representatives to testify. This resulted in two reports that drew on the research and consultations to outline legal and policy issues relevant to HIV (Global Commission on HIV and the Law 2012, 2018).

Based on these findings, programs were launched to implement partnerships with various countries to repeat these analyses at a national level, with resulting action plans. A *Legal Environment Assessment Tool* was used by UN agencies and health officials in numerous countries to review laws and policies relevant to HIV and to convene stakeholders on an agreed program to reform laws and policies, to create an enabling environment for the HIV response (UNDP 2014). Similarly, the Joint UN Programme on HIV (UNAIDS) has convened global partnerships to address stigma, discrimination, and criminalization, and has issued reports and guidance on human rights and HIV, engaging national committees in 38 countries to identify shared programmatic actions to address human rights-related barriers to health.

Taken together, this and other related work in the HIV response has identified diverse political and legal factors that shape health outcomes, promoting an enabling environment in which laws and policies provide support for the funded interventions, rather than undermining them.

The approach taken by HIV agencies and groups also highlights how framing laws and policies as ‘determinants’ may in some respects ‘overdetermine’ external forces, and limit our perception of the potential for human agency. As noted above, the global HIV response shows that individuals can and do work together to change social, economic, and political determinants of health: they could show the way forward for the same kind of work to address those determinants in the digital age.

Learning from this example, perhaps we can put people in a different position in regard to the rainbow. Humans are both inside the arch-shaped by determinants, but also outside, shaping the arch in turn. Through voting, debating, online advocacy, civil society groups, and more, people have and do learn about our rights and use them to mobilize collectively, sharing knowledge and shaping the political determinants of health.

Thus, any visualization of a conceptual framework for political determinants needs to show humans as actively shaping political determinants: as potters, crafting the arches; as athletes, playing with them. Digital governance is something that individuals and communities can interact with and play with to explore a future that better fits our needs.

## CONCLUSION: GOING BEYOND THE RAINBOW

This article has built on previous discussions of digital health and political determinants of inequality in health, proposing a new framework to analyze how political determinants influence digital health, potentially generating either positive or harmful effects. It questions whether integrating ‘digital determinants’ into the socio-economic determinant rainbow model might inadvertently mask the role of politics, in particular, the role of human agency in shaping our own circumstances in the digital age. As an alternative lens, it proposes three categories of political determinants of health in the digital age: laws and policies, governance, and civic engagement; with a transnational reach of private actors and transnational collaborations a through-line considered throughout all three categories of determinant.

This discussion is increasingly pertinent due to the rapid digital transformation of health, particularly accentuated by the COVID-19 pandemic. While WHO’s *Global Strategy for Digital Health (2020-2025)* urged nations to create cohesive digital health strategies, emphasizing strong governance structures to handle the changing landscape where digital access is reshaping health information and services; existing tools are still insufficient for a comprehensive national digital health strategy, as they primarily focus on the health and ICT sectors without considering the broader political, social, and information environment in which people use technology for health.

Drawing lessons from the HIV response, which successfully mobilized legal and policy reforms through systematic multi-stakeholder analyses of legal environments, supported by community engagement and human rights advocacy, this perspective underscores the need for comprehensive national digital health strategies that consider all these political determinants. In particular, it underscores the role of new forms of literacy, knowledge production, and civic engagement in imagining and enacting alternative futures for digital health governance. Unlike the production of rainbows, the governance of digital health is not a naturally occurring phenomenon, and it is one in which the public can play a role in crafting and interacting with political determinants to create a more equitable health landscape.

Like any lens, this proposed approach to political determinants of digital health also has limitations, and future reflection and debate may lead to new approaches. For instance, when asking ‘who is behind the wheel’ of digital determinants that drive inclusion or exclusion in health, some may find the approach proposed here limited or technocratic. Scholars such as Couldry and Mejias (2019), Ferryman (2021), and Sekalala and Chatikobo (2024) have advanced ideological critiques of the digital transformation, arguing that it entrenches existing racialized hierarchies and repeats extractivist colonialities, with only minimal benefits (and potentially real harms) to those in LMIC whose data are extracted to profit private actors in high-income countries. I agree with these critiques, and in the future, LMIC governments and civil society groups may increasingly demand action to address these geopolitical inequalities.

One platform for holding these discussions ought to be the United Nations. In September 2024, the UN General Assembly took the first tentative step towards developing more robust global digital governance by approving the *Global Digital Compact*, which aimed to establish a comprehensive global framework for the governance of digital technology and AI. The *Global Digital Compact* offered new hope for collaboration among UN agencies and member states to promote more robust digital governance; and discussions at the Summit of the Future, where the Compact was launched, emphasized the need to address the digital gender divide, among other inequalities. Critics feel the Compact as finally adopted was weakened (Reiland 2024), and while the Compact did address gender inequalities and reference human rights, it did not mention health (Pugh-Jones 2024).

In 2025, the World Health Assembly is due to review progress on the first *Global Strategy on Digital Health* and decide on the process to develop the next one. It remains to be seen whether WHO will consider the broader political and commercial determinants of digital health in the next strategy, and whether it does or not may depend on future civic engagement.
